# Structures and Strategies for Retaining an International Pediatric Cohort from Birth: Lessons from The Environmental Determinants of Diabetes in the Young (TEDDY) Study

**DOI:** 10.21203/rs.3.rs-4421364/v1

**Published:** 2024-06-06

**Authors:** Patricia Gesualdo, Jessica Melin, Rachel Karban, Claire Crouch, Michael Killian, Diane Hopkins, Annika Adamsson, Joanna Stock, Suzanne Bennett Johnson, Judith Baxter

**Affiliations:** University of Colorado-Anschutz Medical Campus, Aurora, Colorado, United States; Lund University, Malmo, Sweden; University of Colorado-Anschutz Medical Campus, Aurora, Colorado, United States; Pacific Northwest Research Institute, Seattle, Washington, United States; Pacific Northwest Research Institute, Seattle, Washington, United States; Georgia Regents University, Augusta, Georgia, United States; University of Turku, Turku, Finland; Institute of Diabetes Research, Munich, Germany; University of Florida, Tallahassee, Florida, United States; University of Colorado-Anschutz Medical Campus, Aurora, Colorado, United States

**Keywords:** Retention, longitudinal study, type 1 diabetes, pediatric, strategies

## Abstract

**Background:**

Retention of study participants in observational studies is essential to maintaining the representativeness of the population, minimizing selection bias, and assuring sufficient statistical power. The aim of this report is to describe the structures and strategies used to retain participants in The Environmental Determinants of Diabetes in the Young (TEDDY) Study, an observational study of children at increased genetic risk for type 1 diabetes followed in an intense protocol with frequent clinic visits from birth until age 15.

**Methods:**

A systematic review of methodologies used to retain research subjects identified four domains: barrier reduction strategies; community building strategies; follow-up/reminder strategies; and tracing strategies. Independent reviewers categorized the retention strategies implemented by the TEDDY Study into each of these domains. Strategies not fitting into any of these categories were placed into a fifth category unique to TEDDY.

**Results:**

TEDDY identified over one hundred retention strategies used during the 15 years of follow-up; most could be categorized in these domains. Those unique to TEDDY included (1) study organization and structures to support retention; (2) efforts to meet the changing developmental needs of the TEDDY population, (3) implementation of efforts to address protocol challenges in real-time; and (4) employment of a re-engagement protocol for those who had dropped out of the study.

**Conclusion:**

Pediatric cohort studies should include strategies, structures, and resources addressing retention at the study’s initiation. It is recommended that child and parent engagement in addition to the developmental needs of the child be an integrated focus of all strategies. Putting mechanisms in place to address protocol and retention challenges in real time would facilitate effectively addressing challenges as they arise.

**Trial registration:**

ClinicalTrials.gov Identifier: NCT00279318

## Background

Longitudinal cohort studies have the advantage of assessing time-varying relationship between exposures collected over time and the progression of a disease in the absence of treatment (1). However, attrition can threaten the validity of any longitudinal study that seeks to understand the natural history of disease (2). The loss to follow-up can lower the ability of the study to detect true associations existing in a population and the pattern of missing data during follow up may lead to incorrect statistical inference and conclusions concerning the relation between exposure and health outcomes. Participant retention can be challenging, especially in pediatric observational cohort studies where there is no treatment, the study duration is long, and protocols require time and effort from the child and parent. Participant motivation to remain in a study may weaken over time (3). Recommended strategies to promote study retention include using experienced study coordinators, consistency of staff over time, changing strategies depending on the phase of the study, building rapport with families, tailoring strategies to the individual needs of the participant, and engaging the child with communication strategies, activities and incentives that are age-appropriate and change as the child matures (3-8).

The Environmental Determinants of Diabetes in the Young (TEDDY) Study is an international prospective cohort study designed to identify environmental factors and gene-environment interactions that may trigger type 1 diabetes in children genetically at-risk for this disease (9). From July 2004 to February 2010, infants identified with increased genetic risk for type 1 diabetes were enrolled at six centers in four countries-Finland, Germany, Sweden, and the United States, with US centers in Colorado, Georgia/Florida, and Washington. All children were enrolled before 4.5 months and were followed until the development of type 1 diabetes or 15-years of age. The study protocol, designed with an intense follow up of frequent visits and various forms of data collection, presents ongoing retention challenges.

Addressing retention challenges was aided by early analyses to understand reasons why parents refused to enroll, their reasons for staying in the study (10, 11) and factors that predicted early withdrawal (12, 13) Findings from these analyses suggested there were both familial conditions, as well as challenges with a complex and demanding protocol, affecting enrollment and continued participation. These insights enabled the study coordinators to develop tailored interventions to reduce withdrawal (10).

As the TEDDY Study reaches the 20th year since the initial enrollment began, this milestone provides an opportunity to review the successes of retaining this unique pediatric cohort from birth to 15 years of age. This report describes structures and strategies used to retain study participants and highlights innovative strategies currently not found in the literature.

## Methods

### The TEDDY Study

To establish a birth cohort of children at high genetic risk for type 1 diabetes the TEDDY Study initiated a large-scale newborn screening protocol at each of the clinical centers. The screening protocol was a four-step process. Parental consent for genetic testing obtained at or after delivery, included the collection of a cord blood sample or a capillary sample via heel stick. All consenting parents were notified of the result and its meaning. Those meeting the a priori determined eligible genetic criteria were invited to enroll their infant in the follow-up phase of the study. Of the 424,788 infants whose parents consented to the newborn screening, 21,589 were eligible and invited to participate in the follow-up. Of these, 8,667 (40%) enrolled in the study, all having elevated genetic risk for type 1 diabetes. In the population with no known genetic risk or family history of T1D, 1 in 300 are likely to get type 1 diabetes. For those meeting the study’s genetic criteria with no known family history, the risk increases to 3% and for those meeting the genetic criteria and having a first-degree relative with type 1 diabetes, the risk increases to 14%. The study participants are diverse in both demographic characteristics and geographic distribution by design, permitting examination of variable levels of the multiple exposures of interest. The protocol, conducted in five languages (English, Spanish, Finnish, Swedish, and German), included quarterly visits until the age of four years. Children who develop islet autoantibodies, indicating initiation of the autoimmune process associated with type 1 diabetes, continue with quarterly visits. Children who tested negative for islet autoantibodies had their visit frequency reduced to two visits per year after the age of 4. Study visits continue until the child is diagnosed with type 1 diabetes or reaches the age of 15 years. The demands and complexity of the study is best described by the contents of the study visits over time as described in Table 1. Study visits last 1–2 hours and include the collection of various biological samples, a blood draw, clinical measurements, interviews, and questionnaires. The study is approved by local institutional review or ethics boards and is monitored by an External Evaluation Committee formed by the National Institutes of Health (NIH).

### TEDDY Structure

The operational structure of TEDDY reflects the multi-center, multi-cultural, and multi-disciplinary aspects of the study design and research objectives. There are eight scientific committees and a study coordinator committee. All report to the Steering Committee whose voting members are the principal investigators from the clinical centers, the data coordinating center, and the NIH scientific project officer. The primary focus of all committees is scientific inquiry, protocol development, implementation, and data analysis. The committees maintain this focus through participation in monthly conference calls and regular in-person or virtual meetings.

Of importance for study retention, the Study Coordinator Committee (SCC) has been involved with all aspects of protocol development from study initiation, implementation, and protocol evolution over time. The coordinators represent the study nurses, managers, and staff conducting the study visits. They also serve as active members of the scientific committees collaborating with study investigators to resolve any issues with protocol implementation. Having a voice at the table, the coordinators present retention and compliance challenges and solutions at the Steering Committee meetings. The SCC members focus specifically on the participant experience, providing important observations related to participant needs and concerns that are the foundation for successful retention.

Specific processes emerging out of this SCC structure to inform retention include: 1) using data-informed approaches to identify participants at risk for study withdrawal to develop tailored interventions to reduce attrition; 2) assessing retention and study visit compliance with recurrent all-center review of data reports; and 3) sharing cross-center retention strategy development and experience. The aim of these structures and processes are high staff and participant retention characterized by engagement and a collaborative approach that includes participants and their families as partners in research.

### TEDDY Retention

The TEDDY study defines retention as the number of participants enrolled at any given point in time divided by the number still eligible to be followed at that time. Those diagnosed with type 1 diabetes or who died are removed from both the numerator and denominator. When participants actively withdraw from the study, the study staff collect the reason for withdrawal, contact information and permission for future contact. Passive withdrawals are those participants who have not responded to engagement attempts or have not completed any protocol elements over an extended period, but whose contact information is current. Both active and passive withdrawals are re-contacted annually and permitted to rejoin the study. Participants classified as lost-to-follow-up have disengaged from TEDDY and their contact information is inaccurate.

**Figure 1:**
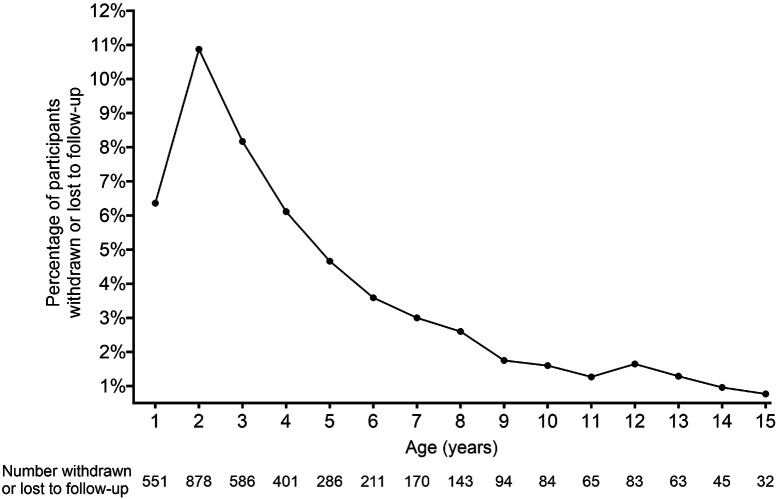
Legend not included with this version.

As of September 30, 2023, 65% of the 8,667 participants enrolled between 2004 and 2010, were still participating in the study (data not shown). Figure 1 describes the percentage of children withdrawn or lost-to-follow-up for the TEDDY cohort by the age of the child. The drop out is highest (11%, *n* = 878) at 2 years of age, with the percentage of children dropping out steadily declining thereafter to rates of less than 2% from age 9 until 15. Among those who actively withdrew from the study, the most common reasons given included distress over the blood draw, the demanding nature of the protocol, and the family being too busy or experiencing stress. Among the 2533 withdrawn, 660 (26%) participants later re-enrolled in the study.

### Retention Strategies Classification

Based on a systematic review of retention strategies among 143 longitudinal cohort studies conducted over the last decade, Teague et al. identified 95 strategies, 44 of which were not previously mentioned in the literature (14). These individual strategies were further classified into four broader categories: barrier-reduction, community-building, follow-up/reminder, and tracing strategies (14). Using meta-regression analyses, the impact on retention of use of these different classes of strategies was assessed.

For this report, study coordinators from the six clinical centers compiled a list of all implemented retention strategies based on reviewing study documentation, retention presentations, and meeting minutes since the initiation of the TEDDY study. Two coordinators independently classified each TEDDY study strategy into one of the four Teague categories. The two independent coding exercises were compared, and the rare discordant results were discussed and reconciled. Several TEDDY retention strategies did not fit into one of the four Teague et al. categories. Consequently, a fifth category of strategies unique to TEDDY was added to the classification system.

## Results

### Retention Strategy Implementation

A summary of the retention strategies employed by the TEDDY study is described in Tables 2A-D. For each Teague category and individual strategy, specific examples used in the TEDDY Study are described. Table 3 describes the strategies unique to the TEDDY Study.

### Teague Domain: Barrier-Reduction Strategies:

Barrier-reduction strategies consist of efforts to support participants in meeting the study protocol’s demands. This includes flexibility in scheduling or location of visits, adapting materials to all relevant languages, and providing childcare or transportation assistance (Table 2A). Each TEDDY center developed specific strategies aimed at making it easier for both children and parents to complete various elements of the protocol. Participant feedback is requested from the parent and child and used to inform retention efforts. Flexibility in the visit schedule, location of the visit and intermittent negotiations in data collection while maintaining fidelity to the protocol is a continual goal.

The approach of creating a personal connection between staff and participants is specific to the clinical center. All TEDDY clinical centers are organized in a way suited to the specific conditions and constraints of their environment. The professional backgrounds of the staff vary significantly by country and center. Some TEDDY centers have multiple staff members managing scheduling and conducting research visits. Other centers opt for a case management approach with families assigned to one dedicated staff member who follow the family for all visits, and were responsible for scheduling, the blood draw, and the data collection. Over time, TEDDY coordinators observed the centers with a case management approach had noticeably higher retention rates. Many of the centers gravitated towards having a hybrid case-management approach where there was a consistent team in contact and an assigned clinical staff for the primary data collection.

### Teague Domain: Community-building Strategies:

Community-building strategies include such things as creating a study logo, gifts with the study logo, and study newsletters (Table 2B). The greatest number of TEDDY retention strategies fall in this domain. A TEDDY logo was used in all study communications, presentations, and annual birthday gifts. As the study participants approached the age of assent, the focus of retention efforts moved away from being exclusively parent to family-child focused engagement strategies. Other strategies used by the TEDDY study in this category were arranging evening meetings with the investigators to learn about study findings, science-focused events for the families, and children had the opportunity to have a pen-pal from another TEDDY clinic.

#### Teague Domain: Follow Up/Reminder Strategies:

This domain includes strategies to encourage participant compliance with study visits and typically involves incentives and various forms of reminders (Table 2C). TEDDY is not unique in the varied uses of incentives as a retention tool. However, there are differences between the US and EU human subjects’ regulations regarding the use of cash incentives. Only the US centers use cash payments. Gift cards, vouchers, and other kinds of financially oriented support are used more broadly, with variability based on local human subject review board considerations.

TEDDY uses multiple forms of communication to keep families engaged by text, email, letters, and phone calls. Keeping up with the family’s mode of contact preferences and utilizing technology effectively requires each center to be both technologically up-to-date and creative.

### Teague Domain: Tracing Strategies:

Tracing strategies involve collecting detailed contact information so the participant can be located even after long periods of absence from the study protocol (Table 2D). In the TEDDY study, the contact information for all primary caregivers and alternate contacts outside of the home is updated at each study visit. The European centers had the additional advantage of country-specific unified registration systems to enable better tracking of participants.

### Unique TEDDY Strategies:

In this category, retention strategies unique to the TEDDY Study are described. These strategies go beyond what has been described in the literature and include study structure to support retention, risk communication and education strategies, addressing challenging protocol elements, data informed approaches to support retention, and the re-engagement protocol (Table 3).

#### Study structure to support retention

refers to the formation of the Child Engagement Committee. The collaboration between the study coordinators and the psychosocial committee resulted in the development of methods for assessing the impact of study participation and factors associated with retention. Together these efforts supported the TEDDY study in being proactive to address challenges as they emerged. The goal was to develop age-appropriate strategies for keeping child participants engaged and informed about the study and type 1 diabetes. The Child Engagement Committee was the force behind developing various child focused engagement strategies including the creation of a series of storybooks and the use of informational videos to connect children in the scientific aspect of the TEDDY study. Initially created by the Swedish coordinators and a professional illustrator, a small picture book, “We Go To TEDDY” was translated into five languages and adopted by all TEDDY clinical centers for the 2–3-year-old children (15). The book used colorful pictures and simple sentences to explain how the character Willie participated in his TEDDY visit. A second storybook and accompanying activity book continued with the story of Will and introduced his classmate Emma, who was also a TEDDY participant. It was developed for school-aged children and was designed to be read together with an adult (16). The book further explained type 1 diabetes, why children were in TEDDY, and how scientists studied the many samples collected in the study. The book referenced a “Junior Scientist” pin, and all study participants were given the same pin. Furthermore, artwork from the book, and other depictions of the same characters were used in study materials and holiday cards. Shortly before the age of 10, a 40 - page chapter book further explaining the type 1 diabetes disease process and a TEDDY child’s risk for developing type 1 diabetes was distributed to the children (17). The same characters, aged to the new target demographic, created continuity and community, while helping children understand their role in the TEDDY study.

#### Risk communication and education strategies

are a result of an early analysis that found inaccurate risk perception was associated with early withdrawal (12). The findings from this analysis led the study coordinators to use varied methods for communicating and assessing the understanding of risk annually with the use of pictographs. This reinforced the importance of study participation for both the parent and child.

Original YouTube informational videos were created and shown at clinic visits, distributed through newsletters, and posted on social media. One video followed the journey of a blood sample from the TEDDY clinic to the study laboratory for processing, and the travel to the large NIH repository for storage (18). Another series of videos used the Junior Scientist illustrations and text to explain diabetes autoimmunity (19). The TEDDY Around the World video, featuring actual TEDDY children, gave a snapshot of each clinical center where the children lived and activities they might do for fun (20). The goal was to emphasize the global span of TEDDY and show children how they were part of something special. Furthermore, it sparked their imagination that TEDDY is much bigger than what they saw at their study visits. Using this video format TEDDY created a unique educational tool, that also served to build community.

#### Addressing challenging protocol elements

includes several of the items in the TEDDY protocol such as the blood draw, stool sample collection, the 3-day food record and the activity meter that were time-consuming and challenging for both parents and children. Several different strategies were developed to make it easier for the families to complete the protocol and to reduce the burden. The staff were trained, both locally and at international meetings, in different methods to reduce anxiety regarding the blood draw. Age-appropriate preparation and distraction methods were developed and used, and other methods for blood collection were presented to the families. The frequency of the stool sample collection was changed. Specific gifts, incentives and age-appropriate information materials were designed to increase the family's motivation to collect stool samples, the 3-day food record and for the child to wear the activity meter.

The creation of the participant portal by the TEDDY Data Coordinating Center highlights an adaptation of technology to improve compliance and reduce a significant barrier to participation. Until this point, all questionnaires required participants to complete paper forms, requiring additional mailings prior to the study visit. Families had to remember to bring the forms to the clinic visit or lengthened the clinic visits to have the forms completed in person. The portal had the advantage of shortening clinic visit time, providing options for the parents, and eventually children, to complete the questionnaires on their personal computer or mobile device, and reducing clinician burden for data entry and follow-up of missing forms.

#### Data-informed approaches to support retention strategies

use study data to develop tailored interventions to optimize retention of the cohort. Reports and scores generated from the data are summarized at the study-wide, regional, center, and participant levels for Coordinators to use as part of their retention strategies.

##### High Risk for Early Withdrawal (HREW) Report

is one example of a cumulative risk model for predicting early withdrawal using demographic data and questionnaire responses collected at screening and at the three-month enrollment visit (12). Nine risk factors were identified, and a risk > 4 classified a child as high risk for early withdrawal (12). All newly enrolled study participants had a risk score calculated and study coordinators were informed of those who scored > 4. The coordinators then designed a tailored intervention specifically for each high-risk family, leading to improvement in study retention (10).

##### Retention-Compliance Score (RCS) Report

is a second example which calculates a standardized score based on the completion of study protocol elements considered to be indicators of exposures, outcomes, and general participation. The RCS score was calculated periodically for all enrolled participants, helping to identify those in need of targeted interventions.

##### The Enrollment Status Report (ESR)

is the primary mechanism for monitoring and measuring retention of the cohort. It describes the enrollment status (enrolled, withdrawn, lost to follow-up, died, and rejoined) and among the enrolled, the degree of activity in the previous 2 years for the total cohort, each clinical center and the site/locations within the center. This report serves to alert centers to changes in withdrawal patterns and level of engagement.

#### Re-engagement protocol,

an additional strategy that sets the TEDDY study apart from other studies, is a specific focus around executing a systematic approach for participants to rejoin the study after a period of withdrawal. A standard cohort surveillance protocol for accurately classifying the study endpoints is leveraged to be a mechanism for maintaining contact information, continual relationship building, and serves as a successful re-engagement retention strategy. All withdrawn participants who agree to future contact are contacted yearly to assess disease status related to study endpoints and to specifically invite families to resume their study participation. Study staff use this approach to communicate that life circumstances, family dynamics, and the TEDDY child’s stage of development could have changed, allowing study participation to become possible again. Approximately 23% of those who withdrew from the TEDDY study later rejoined.

## Discussion

In longitudinal observational study designs, retention must be a central focus for maintaining the representativeness of the population. Minimizing attrition ensures high efficiency when conducting research by maintaining the statistical power to detect associations of interest. Furthermore, reducing attrition can help limit systematic bias that can lead to incorrect estimates of relationships between exposures and health outcomes (2).

Since 2003, the TEDDY Study has been a major investment of the NIH to create and sustain a multi-center collaborative effort designed to establish an international cohort of children at high genetic risk for developing type 1 diabetes. The primary goal is to identify the triggers that may promote, delay, or prevent disease progression. Among the participants enrolled between 2004 and 2010, 65% were retained. Of the enrolled cohort who had previously withdrawn from the study, 660, or 7.6% had re-enrolled. Annual rates of withdrawal and loss to follow-up were highest (11%) during the first two years of participation. As children reached the ages of 5–7 years old, the rate of withdrawal fell from 5–3%. It was at this point, 5 years into the conduct of the study, that enrollment ended, and the coordinator’s focus on retention became a priority. The success of concentrating on retention during this time is evidenced by the rate of withdrawal continuing to decline to less than 2% from the ages of 9 to 15 years.

Establishing an international birth cohort to be followed intensively with a demanding data collection protocol for 15 years, across six centers, meant that the TEDDY study needed to utilize multiple retention strategies. These needed to target both child and family, in such a way as to evolve over time to meet the needs of a developing child and address changing conditions in the family. This study compiled over 100 retention strategies implemented in TEDDY and found similarities in the domains of Barrier Reduction Strategies, Community Building Strategies, Follow-up/Reminder Strategies, and Tracing Strategies published by Teague et al (14). Considerations for retention strategy development in the TEDDY study focused on age-appropriateness, gender, stage of language acquisition, cultural context, study population diversity, and identifying specific participant needs for tailored approaches. These considerations impact everything from selection of clinic décor to the reading level of the newsletters and written materials, to the planning of the most appealing events to celebrate the participants’ contribution to the study. For future pediatric cohort studies wanting to strengthen their retention efforts, strategy development and achievement is most successful when the study structure supports the coordinator role and decision-making surrounding the focus on retention.

Several previous studies presenting retention strategies conclude that financial incentives and barrier-reducing strategies are most effective (6, 14, 21). TEDDY employed a variety of “incentives” but only the US centers provided regular financial incentives. The European centers could not provide financial incentives, yet Sweden had the greatest retention over time (> 70%). This suggests the cultural norms around cash payments need to be taken into consideration. Furthermore, it takes more than cash incentives to keep families engaged and may not be the most effective strategy, especially for a longitudinal study. Other study-branded gifts served as incentives and seem to be more memorable than cash and have the added benefit of promoting community in the cohort. Conventional barrier-reducing strategies such as parking, transportation, and flexible scheduling are important, though not sufficient for more involved research protocols with frequent visits and the collection of multiple data points. The TEDDY study found it important to address barriers arising from the demands of the study protocol by developing a participant portal and the tailoring of the protocol visits to balance the needs of the family and the study. Strategies aimed at reducing barriers also help to guide the options to support families to re-engage to the degree that is realistic for them.

Consistent with recommendations from previous studies, TEDDY has employed experienced coordinators who have established a long-term structure for collaboration permitting them to tailor and experiment with retention approaches to best meet the needs of their center-specific cohorts and individuals (4, 22). In a longitudinal study such as TEDDY, staff consistency and staff retention are critical to fostering and sustaining strong relationships with the child and parent throughout childhood. TEDDY, like the Adolescent Brain Cognitive Development (ABCD) (23) and Maternal Lifestyle Study (MLS) (5) studies, has been proactive in building rapport based on trust and respect, keeping families engaged by anticipating their needs, and being positive about their participation.

Retaining a family is much easier than trying to re-engage one. Recognizing there were many reasons for withdrawal associated with family characteristics not in the study’s control resulted in the TEDDY coordinators instituting the re-enrollment protocol after the screening phase and enrollment was complete. This protocol is an important strategy for long-term engagement and data completeness. In addition, unlike standard longitudinal surveillance protocols that only assess disease status long term, our re-engagement strategies also focus on inviting families back at any time. If a family does withdraw, it is important to find out the reason(s), since knowing these reasons can be a key for future re-engagement. Gaining permission for future contact at the time of withdrawal is critical for re-engagement and the surveillance of study endpoints. In a long-term study, having the ability to check back in after several years when conditions may have changed and providing different options can create a pathway to rejoining. Re-engagement protocols require extensive staff resources, sometimes with a limited return on the overall investment. Although challenging, future studies should ensure the tracking of study participation status, reasons for withdrawal and re-enrollment. This requires a well-designed and fully utilized system that is developed before enrollment begins and implemented consistently. Future analysis is planned to review the data on who rejoins to understand how to best target re-engagement efforts.

In many longitudinal studies, rates of dropout are not uniform, and obvious missing data patterns are important to understand as early as possible so they may be addressed in real-time. It is unfortunate that many studies report drop-out rates or rates of missing data from protocol violations at the time of data analysis instead of using these data to improve retention or protocol compliance on an ongoing basis. TEDDY was fortunate in this regard, as early analyses provided data to inform strategy development (11, 13). Concerns about the blood draw and being too busy to participate were given as reasons for refusing enrollment and later were reasons for withdrawal.(12, 13) Early in the study, the difficulty children and parents had with the blood draws became apparent, and many interventions were put in place to reduce child and parent distress. Similarly, once the factors associated with study drop-out were identified (10), a strategy was put in place to identify those at high risk so these individuals could receive an individually tailored intervention to reduce their risk (13). This avoided employing retention strategies targeted at specific exposures or groups that could have resulted in a systematic bias.

Learning what motivates participants to stay is also important. Based on parent surveys, among those enrolled, 76% had never thought of leaving the TEDDY study, where having someone watching the child for development of type 1 diabetes was the most important reason for staying in the study. Other reasons for staying included helping scientists discover the causes of diabetes, getting the child’s test results from the blood draw, seeing the same staff at TEDDY visits, and the possibility of a prevention trial in the future. A minority (24%) of parents acknowledged some thoughts of leaving TEDDY and cited the blood draws, being too busy/not having enough time, the demanding protocol, and the collection of food records as their reasons for considering leaving. Flexibility around scheduling, visit reminders, free parking, and help with transportation were suggestions to make participation easier (11).

Finally, TEDDY actively changed its communication and engagement strategies to meet the needs of the child’s stage of development and the changing family circumstances. These efforts to use TEDDY data, collected in real-time, to meet the needs and challenges of this unique cohort are critical components of the TEDDY study success in retaining its study cohort over such a long study duration.

However, there were certainly limitations and missed opportunities in the TEDDY study retention strategies. During the first few years of TEDDY, the focus was on screening and enrollment. It was not until after the screening phase was complete that the implementation of the dedicated and innovative retention efforts began. Future studies should emphasize the importance of retention from the beginning with dedicated team members concentrating on specific strategic planning. Another limitation is that the TEDDY study implemented multiple retention strategies simultaneously and with different timing across the clinical centers. As a multi-center study with distinct cultures and constraints, we had to remain flexible in the timing and approach in the implementation of different strategies. While Robinson suggests studies should employ multiple strategies to best improve retention (24), it is difficult to accurately measure the effectiveness of any one strategy at a given point. Fortunately, it was apparent that the dropout of participants continued to decline once the retention strategies were consistently implemented.

## Conclusion

Whether a clinical trial or a longitudinal observational study, cohort retention is critical to the validity and generalizability of the research findings. Without the benefit of randomization, observational studies need to be particularly attentive to the threats arising from attrition and the resulting missing data. The TEDDY study experience supports the recommendations for integrated study coordinator involvement, and the use of multiple strategies informed by data gathered and analyzed for the purposes of implementing targeted, and well considered retention strategies. In pediatric cohort studies, the focus on child and parent engagement is highly recommended. Although it may be difficult to incorporate, research studies need to use data in real-time to inform retention strategies. Future studies should advocate and allocate funds and resources for dedicated retention teams to implement these strategies.

## Figures and Tables

**Table 1:** Data Collected in the TEDDY Study

Data Collected	Frequency by Age
Blood	Every clinic visit*
Stool	Monthly up to 4 years of age; four times a year up to 10 years of age
Tap water	Child-age 9 months; every two years after 3 years of age
Toenails	Child-age 2 years; annually thereafter
Nasal swab	Every clinic visit
Urine	Every clinic visit
Activity Meter	Child-age 5 years; annually up to 10 years of age
Weight and Height	Every clinic visit
Three-day food record	Every 3 months up to 1 year; biannually up to 10 years of age
Parent Questionnaires	Child-age 3, 6, 15, and 27 months; annually thereafter
Child Questionnaire	Child-age 10 years; annually thereafter
TEDDY book extraction	Every clinic visit

* Clinic visits are conducted every 3 months until 48 months and then every 6 months until 15 years. The children who developed islet autoimmunity continued a quarterly schedule until the diagnosis of diabetes or 15 years, whichever came first.

**Table 2:** A-D: Mapping of TEDDY Retention Strategies used 2004–2020 to Meta-Analysis Typology of 4 Strategy Domains and 144 Individual Strategies Developed by Teague, et al^14^

A. DOMAIN: BARRIER REDUCTION	EXAMPLES USED IN TEDDY
With Individual Strategy Listed	
Adapt materials for different languages	*English, Finish, German, Swedish, Spanish added in one US site*
Adjust lab to be more home-like, less clinical	*Age-appropriate child themed décor, playroom/toys*
Assistance with postage costs	*Pre-paid postal and courier for returning questionnaires and lab samples*
Assistance with transport, parking, directions	*Where needed: taxi, ride services, bus fare, parking*
Catering/refreshments	*Standard office refreshments (e.g. Coffee, snacks); lite meal after Oral Glucose Tolerance Test*
Consistency in research staff	*Center-specific approaches included: 1) Assigned clinician as single point of contact for data collection, scheduling, results communication; 2) Participant able to request specific clinician; 3) Consistency in clinical team working with participants*
Extended data collection window	*Changes in data collection frequency -e.g. stool samples from monthly to quarterly,*
Flexibility of research team (e.g., hours called, scheduling)	*Flexible clinic visit times (evenings, weekends)*
Hiring, training, and support of staff	*Centralized training meetings, local training, opportunities for professional development, focus on team development and staff retention*
Matching staff to participants, e.g., by language spoken, nature of questions	*Bilingual (Spanish/English) staff at 1 center that recruited Spanish speaking participants*
Prioritizing measures	*Prioritized endpoint data (blood for autoantibody assay) and exposure data (TEDDY Book) when doing tailored protocol, Blood draw only visits*
Recruiting for long-term retention	*Video describing study used for recruitment and informed consent and re-enrollment*
Simple, efficient procedure	*Combined phone and in-person to make clinic time shorter, more efficient; Abbreviated diet data collection*
Site and home visits	*Home visits, off-site clinics at locations closer to participant’s home, mobile vans, long distance protocol for families who moved away from the clinic area.*
Skip waves	*Protocol Flexibility: Tailored to individual needs when necessary; skipping collection of selected items*
Splitting data collection over multiple sessions	*Used asynchronistic data collection for parent and child*
Toll-free project phone number	*Call routing from centralized number to study smart phones*
Branding	*TEDDY logo used on all materials e.g. presents, questionnaires, brochures, labels*
Certificate of appreciation/completion	*Completion of specific protocol items, Certificate recognizing the child’s halfway point in study at age 7.5 years and at study completion at 15 years.*
Educating the community on research	*Social media, blogs, study-wide and local clinical center websites. Publications in newspapers. Brochures. Talks at public meetings.*
Emphasizing benefits of study	*Knowledge about T1D, symptoms and child’s T1D risk; benefits of early diagnosis; lower risk of ketoacidosis; possibility to participate in prevention studies.*
Events/opportunity to meet other participants	*Science days, museum events, park days, parent/family evening events for study updates and meeting investigators.*
Feedback report	*Study wide parent survey, parent feedback cards, child feedback card using happy to sad faces scale.*
Gift/ freebies	*Birthday presents; gift after blood draw or other protocol items. Coffee, meal after OGTT.*
Hiring, training, and support of staff	*Both center specific training and support, study-wide training meetings for all staff members, regular conference calls*
Letter from chief investigator	*Center specific newsletters yearly to families*
Media coverage	*TV, magazine, news articles that highlighted the TEDDY Study*
Newsletter/e-newsletter	*Newsletters sent to both parents and children separately and center specific.*
Opportunity to participate in other research	*Prevention studies for T1D*
Photo album	*Photos taken at study visit and shared with participant.*
Building rapport	*Conversation log to document the personal events and family milestones outside of study visit to build connection for future interactions.*
Sharing study results	*Results shared on websites, newspapers, social media, newsletters, published article links and lay summaries*
Social media	*Facebook center specific, YouTube, Blogs*
Thank you, birthday, and holiday cards	*Same TEDDY birthday present and holiday cards for all children, center specific birthday and thank you cards.*
Time with chief investigator	*Parents evening events, research doctor available for participants*
Website	*Study-wide and center specific websites to inform participants*and families.
Follow-up brochure	*Materials developed and shared to inform parents of Follow Up study*
Budgeting for multiple contact attempts	*Use of a variety of contact approaches in scheduling and re-engagement protocol*
Extra incentive to complete all data collection points	*US Centers only: variable payments based on completed data items (stool samples and diet records)*
Gift/ freebies incentives (e.g., t-shirts, discount cards)	*Birthday presents; gift after completing blood draw or other protocol items; coupons*
Hiring, training, and support of staff	*Centralized training meetings, local training, opportunities for professional development, focus on team development and staff retention*
Incentive (cash/vouchers)	*US centers only paid participants for completion of different elements of the study protocol*
Incentives raffles/competitions	*Select centers implemented raffles to increase stool sample compliance*
Increased incentive for hard-to-reach	*Some US Centers used additional pay for completing study visit after extended time of not attending*
Limiting number of calls etc. based on participants’ response	*Contact attempts were limited to those who were did not respond*
Medical assistance (e.g., diagnostic testing)	*Results from blood draw and were shared with participant after each visit (e.g., type 1 diabetes, celiac and thyroid disease autoantibodies)*
Phone Follow-up	*Phone calls to discuss positive results*
Provide referrals, e.g., medical or legal	*Referral to specialist for needle phobia /referral to psychologist based on questionnaire response to psychosocial measures*
Email reminder	*Email used for scheduling, study follow up, reminder of visit*
Phone call reminder	*Phone used for scheduling, study follow up, reminder of visit*
Postcard/letter reminder	*Letter sent annually to inactive or withdrawn participants*
SMS reminder	*SMS (text) used for scheduling, study follow up, reminder of visit*
Reminders (unspecified)	*Sent reminders for specific data items multiple times*

**Barrier-Reduction Strategies not used in TEDDY**: Survey design, Schedule two participants simultaneously, pilot testing, minimizing time between data collection points, focus group on survey design, partial data collected from proxy, anonymity for participants, childcare, advisory group, adjusted inclusion criteria

**Community Building Strategies not used in TEDDY**: Champion participants, Gaining support of relevant institutions and organizations, Study membership card

**Follow-up/Reminder Strategies not used in TEDDY**: Incentive increasing value over time; Resend survey once, Resend survey multiple times; SMS follow-up; Website follow-up; Face-to-face reminder (e.g., home visit)

**Table 3:** Retention Strategies Unique to TEDDY with Specific Implementation Examples

D. DOMAIN: TRACING STRATEGIES	EXAMPLES USED IN TEDDY
With Individual Strategy Listed	
Tracing via alternative contacts	*Alternate contact information collected and reviewed at each study visit*
Case-review meetings	*Team review of families challenged by completing study protocol.*
Tracing via change of address cards	*Used return mail option to get a forwarding address for participants who moved.*
Tracing via email	*Attempted to gather multiple emails for household to use to locate*
Hiring, training, and support of staff	*Local staff trained on specific contact approaches*
Tracing via letter	*Letters sent out with return address request to get updated address*
Tracing via phone call	*Multiple phone numbers collected and used to contact*
Tracing via public records	*Use EU registries/online directory (whitepages.com) to update address*
Tracing via tracking database	*Each clinical center used locally designed tracking database*
Tracing via update your details form	*Use of TEDDY Update Form in re-engagement protocol so participants would share current contact information*
UNIQUE TEDDY STRATEGIES	EXAMPLES OF SPECIFIC IMPLEMENTATION
Study structure to support retention	*Coordinator/retention calls, study staff training, Child Engagement Committee, local retention coordinators*
Risk Communication/Education Strategies	*Annual risk conversations, use of pictographs to illustrate risk, Junior Scientist books, child focused informational videos (e.g. Video: What happens to your blood?)*
Addressing challenging protocol elements	*Blood draw interventions including: capillary, finger stick at home or clinic, age-appropriate distractions, expert advisors, staff training to address blood draw anxiety, preparation for child through materials and books, brochures,* *Stool sample: changed frequency of collection, offered specific incentives/rewards* *3-day diet record: more teaching tools to support accurate collection, child friendly/daycare collection tool, added to portal for convenience* *Accelerometers: age-appropriate strategies to increase acceptance and wearing, specific gifts for completion*
Data-informed approaches to support retention	*Implementation of High Risk for Early Withdrawal (HREW) with tailored intervention, Retention-Compliance Score (RCS) Report, Enrollment Status Report (ESR)*
Re-engagement protocol	*Yearly mailed retention materials to re-engage inactive and withdrawn participants, offer of a one-time blood draw.*

**Tracing Strategies not used in TEDDY**: Extensive location tracing information, Tracing via house visit, incentive for staff members, incentive to update contact details, locator form documentation, private investigator, SMS, social media, website, non-public records

## The TEDDY Study Group

**Colorado Clinical Center:** Marian Rewers, M.D., Ph.D., PI^1,4,6,9,10^, Kimberly Bautista^11^, Judith Baxter^8,911^, Daniel Felipe-Morales, Brigitte I. Frohnert, M.D., Ph.D.^2,13^, Marisa Stahl, M.D.^12^, Isabel Flores Garcia, Patricia Gesualdo^2,6,11,13^ Michelle Hoffman^11,12,13^ Randi Johnson, Ph.D.^2,3^, Rachel Karban^11^, Edwin Liu, M.D.^12^, Leila Loaiza, Jill Norris, Ph.D.^2,3,11^, Holly O’Donnell, Ph.D.^8^, Andrea Steck, M.D.^3,13^, Kathleen Waugh^6,7,11^. University of Colorado, Anschutz Medical Campus, Barbara Davis Center for Childhood Diabetes, Aurora, CO, USA.

**Finland Clinical Center:** Jorma Toppari, M.D., Ph.D., PI^¥^1,4,10,13^, Olli G. Simell, M.D., Ph.D., Annika Adamsson, Ph.D.^^11^, Suvi Ahonen^*±§^, Mari Åkerlund^* ±§^, Sirpa Anttila^μ¤^, Leena Hakola, Ph.D.^*±^, Sanni Heikura^μ¤^, Tiia Honkanen ^μ¤^, Heikki Hyöty, M.D., Ph.D.^*±6^, Jorma Ilonen, M.D., Ph.D.^¥3^, Saori Itoshima, M.D.^¥^^, Minna Jokipolvi^*±^ , Sanna Jokipuu^^^, Taru Karjalainen^μ¤^, Leena Karlsson^^^, Jukka Kero, M.D., Ph.D.^¥^3, 13^, Marika Korpela^μ¤^, Jaakko J. Koskenniemi M.D., Ph.D.^¥^^, Miia Kähönen^μ¤11,13^, MikaelKnip, M.D., Ph.D.^*±^, Minna-Liisa Koivikko^μ¤^, Katja Kokkonen^*±^, Merja Koskinen^*±^, Mirva Koreasalo^*±§2^, Kalle Kurppa, M.D., Ph.D.^*±12^, Salla Kuusela, M.D.^μ¤^, Jarita Kytölä^*±^, Mia Laakso^μ¤^, Jutta Laiho, Ph.D.^*6^, Tiina Latva-aho^μ¤^, Siiri Leisku^*±^, Laura Leppänen^^^, Katri Lindfors, Ph.D.^*12^, Maria Lönnrot, M.D., Ph.D.^*±6^, Elina Mäntymäki^^^, Markus Mattila, Ph.D.^*±2^, Maija E. Miettinen, Ph.D.^§2^, Tiina Niininen^±*11^, Sari Niinistö, Ph.D.^§2^, Noora Nurminen^*±^, Sami Oikarinen, Ph.D.^*±6^, Hanna-Leena Oinas^*±^, Paula Ollikainen^μ¤^, Zhian Othmani^¥^, Sirpa Pohjola^μ¤^, Jenna Rautanen^§^, Minna Romo^^^, Juulia Rönkä^μ¤^, Nelli Rönkä^μ¤^, Satu Simell, M.D., Ph.D.^¥12^, Aino Tihinen^μ¤^, Päivi Tossavainen, M.D.^μ¤^, Erika Turtinen^μ¤^, Mari Vähä-Mäkilä^¥^, Eeva Varjonen ^^11^, Riitta Veijola, M.D., Ph.D.^μ¤13^, Irene Viinikangas^μ¤^, Suvi M. Virtanen, M.D.,Ph.D.^*±§2^. ^¥^University of Turku, Turku, Finland, *Tampere University, Tampere, Finland, ^μ^University of Oulu, Oulu, Finland, ^^^Turku University Hospital, Wellbeing Services County of Southwest Finland, Turku, Finland, ^±^Tampere University Hospital, Wellbeing Services County of Pirkanmaa, Tampere, Finland, ^¤^Oulu University Hospital, Wellbeing Services County of North Ostrobothia, Oulu, Finland, ^§^Finnish Institute forHealth and Welfare, Helsinki, Finland.

**Georgia/Florida Clinical Center:** Richard McIndoe, Ph.D., PI^^4,10^, Desmond Schatz, M.D.*^4,7,8^, Diane Hopkins^^11^, Michael Haller, M.D.*^13^, Melissa Gardiner^^11^, Ashok Sharma, Ph.D.^^^, Laura Jacobsen, M.D.*^13^, Percy Gordon^^^, Jennifer Hosford^^^, Sharon Maina^^^. ^^^Center for Biotechnology and Genomic Medicine, Augusta University, Augusta, GA, USA. *University of Florida, Pediatric Endocrinology, Gainesville, FL, USA.

**Germany Clinical Center:** Anette G. Ziegler, M.D., PI^1,3,4,10^, Ezio Bonifacio Ph.D.*, Cigdem Sanverdi, Willi Grätz, Anja Heublein, Sandra Hummel, Ph.D.^2^, Annette Knopff^7^, Melanie Köger, Sibylle Koletzko, M.D.^¶12^, Claudia Ramminger^11^, Roswith Roth, Ph.D.^8^, Jennifer Schmidt, Marlon Scholz, Joanna Stock^8,11,13^, Katharina Warncke, M.D.^13^, Lorena Müller, Christiane Winkler, Ph.D.^2,11^. Forschergruppe Diabetes e.V. and Institute of Diabetes Research, Helmholtz Zentrum München, Forschergruppe Diabetes, and Klinikum rechts der Isar, Technische Universität München, Neuherberg, Germany. *Center for Regenerative Therapies, TU Dresden, Dresden, Germany, ^¶^Dr. von Hauner Children’s Hospital, Department of Gastroenterology, Ludwig Maximillians University Munich, Munich, Germany.

**Sweden Clinical Center:** Åke Lernmark, Ph.D., PI^1,3,4,5,6,8,9,10^, Daniel Agardh, M.D., Ph.D.^6,12^, Carin Andrén Aronsson, Ph.D.^2,11,12^, Rasmus Bennet, Corrado Cilio, Ph.D., M.D.^6^, Susanne Dahlberg, Malin Goldman Tsubarah, Emelie Ericson-Hallström, Lina Fransson, Emina Halilovic, Susanne Hyberg, Berglind Jonsdottir, M.D., Ph.D.^11^, Naghmeh Karimi, Helena Elding Larsson, M.D., Ph.D.^6,13^, Marielle Lindström, Markus Lundgren, M.D., Ph.D.^13^, Marlena Maziarz, Ph.D., Jessica Melin, Ph.D.^11^, Kobra Rahmati, Anita Ramelius, Falastin Salami, Ph.D., Anette Sjöberg, Evelyn Tekum Amboh, Carina Törn, Ph.D.^3^, Ulrika Ulvenhag, Terese Wiktorsson, Åsa Wimar^13^. Lund University, Lund, Sweden.

**Washington Clinical Center:** William A. Hagopian, M.D., Ph.D., PI^1,3,4,6,7,10,12,13^, Michael Killian^6,7,11,12^, Claire Cowen Crouch^11,13^, Jennifer Skidmore^2^, Trevor Bender, Megan Llewellyn, Cody McCall, Arlene Meyer, Jocelyn Meyer, Denise Mulenga^11^, Nole Powell, Jared Radtke, Shreya Roy, Preston Tucker. Pacific Northwest Research Institute, Seattle, WA, USA.

**Pennsylvania Satellite Center:** Dorothy Becker, M.D., Margaret Franciscus, MaryEllen Dalmagro-Elias Smith^2^, Ashi Daftary, M.D., Mary Beth Klein, Chrystal Yates. Children’s Hospital of Pittsburgh of UPMC, Pittsburgh, PA, USA.

**Data Coordinating Center:** Jeffrey P. Krischer, Ph.D., PI^1,4,5,9,10^, Rajesh Adusumali, Sarah Austin-Gonzalez, Maryouri Avendano, Sandra Baethke, Brant Burkhardt, Ph.D.^6^, Martha Butterworth^2^, Nicholas Cadigan, Joanna Clasen, Ph.D., Kevin Counts, Laura Gandolfo, Jennifer Garmeson, Veena Gowda, Shu Liu, Xiang Liu, Ph.D.^2,3,8,13^, Kristian Lynch, Ph.D.^6,8^, Jamie Malloy, Lazarus Mramba, Ph.D.^2^, Cristina McCarthy^11^, Hemang M. Parikh, Ph.D.^3,8^, Cassandra Remedios, Chris Shaffer, Susan Smith^11^, Noah Sulman, Ph.D., Roy Tamura, Ph.D.^1,2,11,12,13^, Dena Tewey, Henri Thuma, Michael Toth, Ulla Uusitalo, Ph.D.^2^, Kendra Vehik, Ph.D.^4,5,6,8,13^, Ponni Vijayakandipan, Melissa Wroble, Jimin Yang, Ph.D., R.D.^2^, Kenneth Young, Ph.D. *Past staff: MichaelAbbondondolo, Lori Ballard, Rasheedah Brown, David Cuthbertson, Stephen Dankyi, Christopher Eberhard, Steven Fiske, David Hadley, Ph.D., Kathleen Heyman, Belinda Hsiao, Christina Karges, Francisco Perez Laras, Hye-Seung Lee, Ph.D., Qian Li, Ph.D., Colleen Maguire, Wendy McLeod, Aubrie Merrell, Steven Meulemans, Jose Moreno, Ryan Quigley, Laura Smith, Ph.D.* University of South Florida, Tampa, FL, USA.

**Project scientist:** Beena Akolkar, Ph.D.^1,3,4,5,6,7,9,10^. National Institutes of Diabetes and Digestive and Kidney Diseases, Bethesda, MD, USA.

**Other contributors:** Thomas Briese, Ph.D.^6^, Columbia University, New York, NY, USA. Todd Brusko, Ph.D.^5^, University of Florida, Gainesville, FL, USA. Teresa Buckner, Ph.D.^2^, University of Northern Colorado, Greeley, CO, USA. Suzanne Bennett Johnson, Ph.D.^8,11^, Florida State University, Tallahassee, FL, USA. Eoin McKinney, Ph.D.^5^, University of Cambridge, Cambridge, UK. Tomi Pastinen, M.D., Ph.D.^5^, The Children’s Mercy Hospital, Kansas City, MO, USA. Steffen Ullitz Thorsen, M.D., Ph.D.^2^, Department of Clinical Immunology, University of Copenhagen, Copenhagen, Denmark, and Department of Pediatrics and Adolescents, Copenhagen University Hospital, Herlev, Denmark. Eric Triplett, Ph.D.^6^, University of Florida, Gainesville, FL, USA.

Committees:

^1^Ancillary Studies, ^2^Diet, ^3^Genetics, ^4^Human Subjects/Publicity/Publications, ^5^Immune Markers, ^6^Infectious Agents, ^7^Laboratory Implementation, ^8^Psychosocial, ^9^Quality Assurance, ^10^Steering, ^11^Study Coordinators, ^12^Celiac Disease, ^13^Clinical Implementation.

## Abbreviations

ESR: Enrollment Status Report
HREW: High Risk for Early Withdrawal
NIH: National Institutes of Health
RCS: Retention-Compliance Score Report
SCC: Study Coordinator Committee
T1D: Type 1 Diabetes
TEDDY: The Environmental Determinants of Diabetes in the Young
